# Editorial: Implementation of physical, psychosocial, and mind-body approaches for the management of osteoarthritis

**DOI:** 10.3389/fresc.2022.1023931

**Published:** 2022-09-12

**Authors:** Shabana Amanda Ali, Jocelyn L. Bowden

**Affiliations:** ^1^ Bone and Joint Center, Department of Orthopaedic Surgery, Henry Ford Health, Detroit, MI, United States; ^2^ Center for Molecular Medicine and Genetics, School of Medicine, Wayne State University, Detroit, MI, United States; ^3^ Department of Physiology, College of Human Medicine, Michigan State University, East Lansing, MI, United States; ^4^ Sydney Musculoskeletal Health, Kolling Institute, The University of Sydney, Sydney, NSW, Australia; ^5^ Department of Rheumatology, Royal North Shore Hospital, Sydney, NSW, Australia

**Keywords:** implementation science, disease management, precision medicine, person-centered, care, intervention

**Editorial on the Research Topic** Implementation of physical, psychosocial, and mind-body approaches for the management of osteoarthritis By Ali SA and Bowden JL. (2022) Front. Rehabilit. Sci. 3: 1023931. doi: 10.3389/fresc.2022.1023931

## Introduction

Osteoarthritis is a debilitating chronic condition estimated to impact 650 million people over age 40 worldwide (1). Painful joints compromise normal function to significantly reduce quality-of-life, yet there is still no cure for osteoarthritis. Clinical practice guidelines recommend physical (e.g., exercise), psychosocial (e.g., cognitive behavioral therapy), and mind-body (e.g., yoga) approaches as effective strategies for osteoarthritis management (2, 3), though there remain barriers to implementing these interventions in clinical settings, creating an evidence-practice gap in osteoarthritis care. Novel implementation methods are needed to ensure the right care is provided at the right time to the individuals who will benefit most. To meet this need, our Frontiers Research Topic focuses on strategies to better implement physical, psychosocial, and mind-body approaches into osteoarthritis management. Contributions were received on a range of topics with a common theme emerging surrounding the need for more tailored approaches—also known as precision medicine, or person-centered care. In this Editorial we briefly review each of the contributions, including two review articles and two perspective articles, and summarize key take-home messages from our Research Topic.

## Delivering evidence-based osteoarthritis care

While the benefits of “core” recommended lifestyle and behavioral approaches including “exercise, physical activity, weight management, and education to support self-management” in osteoarthritis are understood (4), successful translation of these approaches from the clinical trial to real-world delivery are lagging. Similarly the link between inpatient, outpatient, and community-based osteoarthritis care is inconsistent across contexts. Our authors have provided two articles describing how thinking outside the box (e.g., the formation of stronger local networks) might improve uptake of best-practice osteoarthritis care in different settings.

Work led by Dr. Julia Chevan aimed to embed the US Arthritis Foundation's “Walk with Ease” program into the post-graduate Physical Therapy curriculum at Springfield College, USA. The authors provide their perspectives on how the implementation of this program delivered several benefits by: (i) providing a much needed community-based physical activity program to local people with osteoarthritis, (ii) enhancing the student experience in providing osteoarthritis care, including health coaching and behavior change models, and (iii) increasing engagement of rehabilitation professionals in the delivery of evidence-based osteoarthritis health programming. Our authors further reflect on how embedding such public health programs into post-graduate curriculum has enriched the student experience by supporting them to deliver tailored, person-centered care, guided by the person with osteoarthritis. The students involved reflect on how the program was critical to their growth as future rehabilitation professionals, and has enshrined new perspectives on the different joys and challenges of exercising with osteoarthritis. This innovative, low-cost, and locally delivered health strategy illustrates how high-value osteoarthritis care can be implemented outside formal health care settings to deliver multi-level benefits to the community.

Delivery of high-value osteoarthritis care is also a challenge in areas with lower resourced health systems (5). The burden of osteoarthritis in low- and middle-income countries, for example, is rapidly rising (6), yet little is known about the determinants of osteoarthritis health or how best to tackle the problem. Dr. Jillian P. Eyles co-authored a mini-review with Drs. Sharma, Weiss Telles, Namane, Hunter, and Bowden, seeking to better understand the challenges in implementing high-quality osteoarthritis care in low- and middle-income countries, including three of the authors' home countries of Nepal, Brazil, and South Africa. The authors review the current knowledge around the barriers to implementation and provide thoughtful discussion on existing opportunities within the different health systems.

## Towards precision medicine for osteoarthritis

A major challenge in the osteoarthritis field is that a “one size fits all” intervention inevitably fails to mitigate the heterogeneity in symptoms (e.g., pain), function (e.g., mobility), and structure (e.g., joint damage) observed across individuals (7). This failure warrants the development and implementation of more tailored interventions that address the individual needs of people with osteoarthritis, for example by accounting for prominent risk factors such as age and sex. Our authors have provided a timely review on the use of hormone replacement therapy in older women with respect to outcomes for osteoarthritis and cardiovascular disease, as well as an insightful perspective article describing the potential for genetics to guide exercise prescriptions for osteoarthritis.

Recognizing that osteoarthritis is more frequently found in women vs. men over the age of 50 years, a narrative review led by Dr. Baraa K. Al-Khazraji explored the roles of menopause and the effects of hormone replacement therapy in modifying outcomes for both osteoarthritis and cardiovascular disease. This article exemplifies the utility of examining specific subgroups of osteoarthritis patients—here, older women—wherein tailored interventions such as hormone replacement therapy may be particularly beneficial. Presenting data from both animal and human studies, the authors summarize different types of hormone replacement therapies, potential mechanisms of action, effects on disease risk and outcomes, and future research directions to better understand and implement this intervention.

Despite abundant evidence to support exercise as a first-line intervention for osteoarthritis (8), there remain challenges with implementation. A perspective article co-led by Drs. Osvaldo Espin-Garcia and Shabana Amanda Ali suggest this may be due in part to the lack of clarity in determining which patients will benefit most from which exercises. The authors propose that genetics can be used to guide exercise prescriptions for specific patient populations, thereby championing a precision medicine approach in osteoarthritis care. They suggest polygenic risk scores applied to early or at-risk osteoarthritis populations can inform prescription of aerobic, neuromuscular, and resistance exercises that will most benefit specific subgroups. As of now, the authors point to a paucity of studies at the intersection of genetics, exercise, and osteoarthritis, and highlight opportunities for future research to advance this field.

## Summary

The collection of papers in this Research Topic highlights the breadth of implementation research being undertaken for osteoarthritis, as well as areas of opportunity. Physical, psychosocial, and mind-body approaches to osteoarthritis management have long been viewed as “lesser” options compared to surgical and pharmacological therapies. However, as the authors in this Research Topic clearly argue, better integration of these approaches into osteoarthritis management is essential to providing the precision, person-centered care needed to reduce the burden of osteoarthritis internationally. It is exciting to see discussion of issues in a “real world” context, outside the realm of efficacy and effectiveness trials. We thank our authors for providing much food for thought by describing novel possibilities to better implement physical, psychosocial, and mind-body approaches into osteoarthritis care.

## References

[1] Cui A Li H Wang D Zhong J Chen Y Global LH. Regional prevalence, incidence and risk factors of knee osteoarthritis in population-based studies. EClinicalMedicine. (2020) 29–30:100587. 10.1016/j.eclinm.2020.100587 PMC7704420 34505846

[2] Bannuru RR Osani MC Vaysbrot EE Arden NK Bennell K Bierma-Zeinstra SMA Oarsi guidelines for the non-surgical management of knee, hip, and polyarticular osteoarthritis. Osteoarthritis Cartilage. (2019) 27(11):1578–89. 10.1016/j.joca.2019.06.011 31278997

[3] Kolasinski SL Neogi T Hochberg MC Oatis C Guyatt G Block J 2019 American college of rheumatology/arthritis foundation guideline for the management of osteoarthritis of the hand, hip, and knee. Arthritis Rheumatol. (2020) 72(2):220–33. 10.1002/art.41142 31908163 PMC10518852

[4] Bowden JL Hunter DJ Deveza LA Duong V Dziedzic KS Allen KD Core and adjunctive interventions for osteoarthritis: efficacy and models for implementation. Nat Rev Rheumatol. (2020) 16(8):434–47. 10.1038/s41584-020-0447-8 32661322

[5] Hunter DJ March L Chew M. Osteoarthritis in 2020 and beyond: a lancet commission. Lancet. (2020) 396(10264):1711–2. 10.1016/S0140-6736(20)32230-3 33159851

[6] Yahaya I Wright T Babatunde OO Corp N Helliwell T Dikomitis L Prevalence of osteoarthritis in lower middle- and low-income countries: a systematic review and meta-analysis. Rheumatol Int. (2021) 41(7):1221–31. 10.1007/s00296-021-04838-y 33907879 PMC8164595

[7] Deveza LA Melo L Yamato TP Mills K Ravi V Hunter DJ. Knee osteoarthritis phenotypes and their relevance for outcomes: a systematic review. Osteoarthritis Cartilage. (2017) 25(12):1926–41. 10.1016/j.joca.2017.08.009 28847624

[8] Verhagen AP Ferreira M Reijneveld-van de Vendel EAE Teirlinck CH Runhaar J van Middelkoop M Do we need another trial on exercise in patients with knee osteoarthritis? No new trials on exercise in knee OA. Osteoarthritis Cartilage. (2019) 27(9):1266–9. 10.1016/j.joca.2019.04.020 31220609

